# Directions for improving the financial support of forensic expert forensic activity in Ukraine with account of positive foreign experience

**DOI:** 10.1016/j.fsisyn.2024.100565

**Published:** 2024-12-11

**Authors:** Nataliia Martynenko

**Affiliations:** Department of Political Sciences and Law, Kyiv National University of Construction and Architecture, 31, Povitroflotsky Avenue, Kyiv, 03037, Ukraine

**Keywords:** Financial support, Forensic expert, Financial statements, Forensic examination, Financial monitoring, Specialized state forensic institutions, Forensic expert activity

## Abstract

The relevance of the issues studied in this article is due to the need to form an efficiently functioning system of regulating forensic expert activity in general and its individual components, in particular, financial support, taking into account positive foreign experience. The author proposes to introduce separate budget programmes for state specialised institutions of each state agency, which will help to resolve the issues of proper funding and justification of expenditures, and will serve as an anti-corruption factor. Attention is focused on the need to develop a unified methodological approach to calculating the number of forensic examinations. The author emphasises the importance of summarizing the financial statements of state specialised institutions engaged in forensic activities and making them available to the public. It is stated that there is an urgent necessity to find reasonable opportunities to expand the financial autonomy of state specialised institutions. The author proves the need to include forensic experts in the list of persons obliged to file declarations and introduce financial monitoring of forensic experts’ lifestyle. The author concludes that it is expedient to introduce a mixed system for determining the cost of forensic examinations.

## Highlights

• The soundness of forensic examination costs depends on the reasonableness of the cost of an expert hour.
• A common methodological approach to calculating the number of forensic examinations standardizes their accounting.
• Summarizing the financial statements of departmental state specialised institutions engaged in forensic expert activities will contribute to the effectiveness of administrative decisions.
• A proper funding will be facilitated by the introduction of separate budget programmes for state specialised institutions of each state agency.
• Introduction of financial monitoring of the lifestyle of forensic experts will allow the implementation of effective corruption prevention mechanisms.

## 1 Introduction

The reform of the judicial system is currently underway in Ukraine. Forensic science being one of that system's important constituent parts, the state and modern science should urgently address new tasks facing it. The actual system of legal acts regulating forensic expert activity in Ukraine is extensive, and the social relations requiring legal regulation are complex and specific. Recently, there has been a pressing need to improve forensic science legislation, which necessitates consideration of the essence and specifics of financial support for forensic science.

In the system of ensuring forensic expert activity, there are various important areas that affect the quality and effectiveness of forensics: scientific and theoretical, regulatory, organisational and administrative, personnel, methodological, material and technical, informational, and financial support. Neglect and disregard of at least one of these components can lead to a significant reduction in the possibilities of using scientific and intellectual tools to ensure the efficiency of justice. This confirms the relevance of discussions around the problems of ensuring forensic expert activity, which, like many other areas of state regulation, requires periodic verification and reform. Given the significant role of forensic science, it is extremely important to establish an efficiently functioning system of its regulation both in general and its individual components, such as financial support, in particular.

In Ukraine, forensic expert activities are carried out professionally by state specialised institutions of six agencies – the Ministry of Justice of Ukraine, the Ministry of Health of Ukraine, the Ministry of Internal Affairs of Ukraine, the Ministry of Defence of Ukraine, the Security Service of Ukraine, and the State Border Guard Service of Ukraine – as well as by forensic experts who are not employees of these institutions, and other specialists in the relevant fields of knowledge [1,2].

Forensic expert activity is the activity of public authorities, legal entities and individuals to ensure the justice of Ukraine with independent, qualified and objective forensic expertise performed with the maximum use of the achievements of science, technology, art and craft [3].

State specialised institutions carry out forensic examinations in criminal proceedings on behalf of investigators, coroners, prosecutors, courts and in cases of administrative offences at the expense of funds allocated for the purpose from the State Budget of Ukraine [1].

Forensic medical and forensic psychiatric institutions carry out forensic examinations, investigations and studies at the expense of funds allocated from the State or local budget [1].

In civil and commercial cases, the costs of forensic examinations are reimbursed in accordance with the procedure provided for by applicable law [1].

State specialised institutions, forensic medical and forensic psychiatric institutions conduct forensic examinations, investigations and studies in criminal proceedings at the request of a suspect, accused, convicted, acquitted person, their defence counsel, legal representative, victim, or his/her representative at the expense of the commissioner [1].

State specialised institutions, as well as experts who are not employees of these institutions, perform other work on a contractual basis [1].

Experts are paid remuneration for the work performed if that work is not their official duty, the amount of remuneration not exceeding the standard cost of the relevant types of forensic examination in the research institutions of the Ministry of Justice of Ukraine [4].

It should be emphasized that funding of the state specialised institutions of the Ministry of Internal Affairs of Ukraine, the Ministry of Defence of Ukraine, the Ministry of Health of Ukraine, the Security Service of Ukraine and the State Border Guard Service of Ukraine is carried out in accordance with the budgets prepared by the management of the relevant ministries and services. The websites of these agencies do not give information on the financial support of forensic activities.

The funding under a separate budget programme is provided only for the forensic research institutions of the Ministry of Justice of Ukraine. Therefore, it is proposed to conduct a study of the financial support of forensic research institutions of the Ministry of Justice of Ukraine.

The purpose of the study is to analyse the trends in the financial support of forensic activities in Ukraine, to identify the main problems, to clarify their essence and causes, and to propose ways to solve them with due regard for foreign experience.

## 2 Literature review

Various aspects of financial support for state-owned specialised forensic institutions have been the subject of a number of scientific studies. Some Ukrainian researchers believe that an adequate financial support for the activities of forensic institutions is the basis for a proper discharge of their functions and undertaken duties [5], acting as an anti-corruption safeguard, and allowing them to attract qualified personnel [6]. The problems related to the financial support of expert activities specifically include the formation of expert services cost, an unresolved issue of the cost of private experts’ work, and some others [7,8]. The legal framework for forensic expert activity in Ukraine is marked by certain significant gaps and shortcomings, which does not guarantee the independence of a forensic expert [9]. Both the scientific community and forensic practitioners expect the adoption of the Law “On Forensic Expertise in Ukraine” and creation of conditions for an effective expert support of justice, enabling forensic experts of state institutions as well as private experts to work honestly, transparently, in competition with other professionals, taking into account the national specificity and international standards [10].

The study of financial support for state specialised institutions is of particular importance due to the specifics of forensic science. Well-known scholars rightly point out that strategic planning of resources that are scarce should focus on the role of a forensic laboratory in the justice system [11]. The economic interpretation of the mission of the forensic laboratory helps in analysing public funding and the return from state investment in it [12].

When investing budgetary funds in the development of state expert institutions, it would be illogical for the state to ‘nurture them as art for art's sake’ without expectations of selfless service to society in the form of proper high-quality provision of expert opinions to the judiciary [13]. The forensic laboratory serves justice, which requires sufficient funding, staffing, and facilities to conduct investigations and provide opinions in a timely manner to ensure public safety [14]. There can be no question of a decline in quality in forensic science, despite funding problems and the growing demand for forensics [15,16,17,18,19]. Taking quality indicators into account will allow for efficient resource allocation and avoidance of unnecessary costs [20]. The introduction of new methods and technological capabilities helps to solve funding-related problems [21,22].

At the same time, a number of issues remain unresolved. The opinions expressed by scientists regarding financial support and the range of laws that form its basis are diverse and inconsistent, which negatively affects the development of forensic science. These considerations once again demonstrate the need for further independent research of the problems arising in this area.

## 3 Results

Under the Ministry of Justice of Ukraine, forensic activities are performed by seven state-owned specialised institutions, namely: Kyiv Scientific Research Institute of Forensic Expertise, National Scientific Centre “Hon. Prof. M.S. Bokarius Forensic Science Institute”, Odesa Scientific Research Institute of Forensic Expertise, Lviv Scientific Research Institute of Forensic Expertise, Dnipro Scientific Research Institute of Forensic Expertise, Research Centre of Forensic Expertise in the field of Information Technology and Intellectual Property, and Research Centre of Independent Forensic Expertise [2].

The Ministry of Justice of Ukraine organises scientific and methodological support for forensic expert activities, performs organisational and managerial functions in relation to forensic research institutions under its authority, determines the procedure and exercises control of compliance with the legislation on forensic activities by these research institutions [23].

Budgetary funding of the forensic research institutions of the Ministry of Justice of Ukraine is based on the main categories of the Budget Code of Ukraine, such as the cost estimate, targeted programme method in the budget process, and the budget programme passport.

The cost estimate is the main document of financial planning of a budgetary institution, which establishes its authority for the budget period to receive revenues and allocate budgetary funds for budgetary obligations and payments in order to perform its functions and achieve the results determined in accordance with the budget allocations [24].

The cost estimate has the following components: the general fund, which contains the amount of revenues from the general fund of the budget and the distribution of expenditures according to the full economic classification of budget expenditures for the performance of the budgetary institution's main functions or the distribution of loans from the budget according to the classification of budget lending; the special fund, which contains the amount of revenues from the special fund of the budget for a specific purpose and their distribution according to the full economic classification of budget expenditures for the implementation of relevant expenditures in accordance with the law, as well as for the implementation of priority measures related to the performance of the institution's core functions, or the distribution of budgetary loans in accordance with the legislation by the classification of budgetary loans [24].

The funding of the forensic research institutions of the Ministry of Justice of Ukraine is provided under a separate budget programme CPCEL 3601070 “Conducting Forensic Examination and Development of Forensic Methodology” using the targeted programme method. A budget programme is a set of measures aimed at achieving a single goal, tasks and expected result, the definition and implementation of which is carried out by the budgetary funds administrator in accordance with the assigned functions [24]. The purpose of the budget programme CPCEL 3601070 “Forensic Examination and Development of Forensic Methodology” is to conduct forensic examinations for the inquiry, pre-trial investigation, and judicial authorities and to develop methods and research techniques in the field of forensic examination, which are implemented in forensic practice with due regard for modern achievements of science and technology [25].

The peculiarity of budget planning is that the amount of funding is planned for the entire budget period. The amount of planned expenditures is determined depending on the number of expected examinations.

The cost of one expert hour is adjusted annually as of January 1, taking into account the consumer price index (inflation index) for the relevant period [4].

Every year, the standard cost of one expert hour is approved by departmental regulations. In 2020, it amounted to USD 6.90 [[26], [27], [28], [29]]; in 2021 – USD 6.10 [[30], [31], [32], [33]]; in 2022 – USD 6.92 [[34], [35], [36], [37]]; and in 2023 – USD 6.54 [[38], [39], [40], [41]].

In order to ensure that the standard cost of one expert hour is in line with the actual cost of conducting an expert examination by state forensic institutions, a resolution of the Cabinet of Ministers of Ukraine of December 5, 2023 stipulated that the cost of expert examinations ordered in criminal proceedings and cases of administrative offences, and studies based on criminal proceedings ordered by pre-trial investigation, prosecution and court authorities, shall be determined in accordance with the standard cost of one expert hour in state specialised forensic institutions, which is USD 9.96, taking into account the actual time spent on forensic examination [4,42,45]. In the Expert Service of the Ministry of Internal Affairs of Ukraine and the Main Forensic Centre of the State Border Guard Service of Ukraine, with account of the consumer price index for 2023, the standard cost of one expert hour in 2024 is USD 10.47 [[43], [44], [45]].

Thus, the cost of one expert hour increases annually: in 2022 compared to 2021 – by 1.13 times, and in 2024 compared to 2023 – by 1.6 times.

Due to the insufficient justification of this indicator, it is currently difficult to verify the validity of the determined cost of each examination. Some expert institutions and experts, in order to cover the real cost of the examination, taking into account all the costs incurred in connection with it, simply convert the cost of the examination into the required number of hours or even artificially increase the number of hours required for an examination, or resort to splitting one examination into several separate ones, which leads to an unduly increase in the number of examinations conducted.

The forensic research institutes of the Ministry of Justice of Ukraine differ in their methods of calculating the number of examinations: by the number of research objects; by the number of forensic experts involved in an examination; in accordance with the expert task set by the customer of the examination, etc.

In Germany, if an expert provides services in several subject areas for which different hourly rates are set, the fee is calculated evenly for the entire required time according to the highest hourly rate [46]. If the calculation of the fee would lead to an unfair result, given the nature of the service, the hourly rate is determined at a reasonable discretion [46].

In Ukraine, there is neither a unified methodological approach to calculating the number of forensic examinations nor a commonly accepted standard for calculating their cost.

State specialised institutions are usually unable to justify for their customers the different costs of examinations that are similar in terms of expert task and complexity [8]. According to the Odesa Scientific Research Institute of Forensic Expertise of the Ministry of Justice of Ukraine, the amount of time spent on an expert examination can range from 6 to 1300 h. A forensic expert spends 1–3 h on preparatory activities for a particular examination alone, and sometimes 8–16 expert hours in particularly complex examinations for preliminary review of provided materials, drafting a motion, determining the required number of expert hours, etc [8]. There is no generally accepted system for calculating expert hours either in state specialised institutions or among private forensic experts.

The CEPEJ Guidelines set out that the remuneration of a court-appointed expert may be measured by hourly rates which depend on specialisation and take into account the expert's qualifications [47].

Some Ukrainian scholars believe that, when establishing the cost of an expert examination, it is advisable to take into account the length of service and the coefficient for the expert's academic degree [8].

In the Slovenian Republic, when determining the cost of a forensic examination, the following is assessed: the number of sheets of the court file and additional documents to be examined; each hour spent examining the objects of study at the crime scene or location, within the limit of the amount; a written opinion and an additional written opinion for which three levels of complexity are defined; each of the started half hour: for the time spent travelling to and from the examination, for the time spent travelling to and from the court, waiting for the hearing, and for oral presentation [48]. The court determines the complexity of the examination and the written opinion [48].

In the Czech Republic, the remuneration for the performance of a forensic examination is regulated by an agreement with the commissioner. If the commissioner is a public authority, the remuneration is determined in accordance with the legal provisions. A list of actions for which an expert is entitled to a fixed remuneration and its amount are approved. For each hour spent performing expert work outside the list, experts are entitled to remuneration. Conditions for raising or reducing the expert's fee, or a complete refusal to pay in case of a poor quality of examination are established. The reimbursement of out-of-pocket expenses and compensation for loss of time, including travelling, is detailed. The list of out-of-pocket expenses for which experts are entitled to fixed compensation and the amount of compensation for out-of-pocket expenses are approved [49,50].

In Germany, the fee of a forensic expert is determined with reference to the hourly rate in the relevant subject area. The decision to engage a expert is determined by attributing the service to a particular subject matter area. The expert's fee is increased by 20 per cent if at least 80 per cent of the services are provided between 23:00 and 6:00 or on Sundays or public holidays [46].

A positive aspect for the financial support of state specialised institutions is the possibility of providing paid services, which allows litigants to order the necessary forensic expertise. The cost of forensic examinations and expert studies included in the list of paid services provided by the forensic research institutions of the Ministry of Justice of Ukraine is determined in accordance with the standard cost of one expert hour [4,51]. The own revenues of budgetary institutions are received in addition to the general budget funds and are included in the special budget fund [24]. The own revenues of budgetary institutions are revenues received in accordance with the established procedure as payment for the provision of services, performance of works and targeted activities, grants, gifts and charitable contributions, as well as revenues from the sale of products or property and other activities in compliance with the established procedure [24].

The forensic research institutions of the Ministry of Justice of Ukraine provide the following paid services:

- forensic examinations, studies and investigations in criminal proceedings at the request of a suspect, accused, convicted, acquitted person, their defence counsel, legal representative, victim, his/her representative and legal representative;
- forensic examinations in civil and commercial cases, as well as cases of administrative jurisdiction;
- at the request of individuals or legal entities, expert studies with the use of forensic tools and methods; consultations requiring specialised knowledge;
- training and internships of specialists who are not employees of state specialised institutions for the purpose of awarding or confirming the qualification of a forensic expert in accordance with the procedure established by the Law of Ukraine “On Forensic Examination”, as well as internships of foreigners and stateless persons;
- scientific research, scientific and technical expertise;
- preparation, publication and distribution of methodological, scientific and methodological, scientific, scientific and technical publications, printed and e-periodicals;
- scientific and practical seminars, conferences, symposia on the theory and practice of forensic examination and criminalistics;
- training of highly qualified scientific personnel;
- advanced training of specialists;
- expert reviews of project documentation for the construction of facilities in accordance with the law;
- assessment of compliance with the requirements of technical regulations in accordance with the law;
- evaluation of property, property rights, expert monetary valuation of land plots and rights to them, as well as reviewing reports on such valuation [51].

The above services are provided by the forensic research institutions of the Ministry of Justice by mutual consent of the parties for a fee that reimburses the costs of their performance.

Thus, the subjects of financial support for forensic expert activity are, on the one hand, the state – allocation of funds from the State Budget of Ukraine, and, on the other hand, the requestor of the examination – at their own funds (see Table 1).

**Table 1:** The structure and scope of financial support for forensic research institutions of the Ministry of Justice of Ukraine.

№	Indicators	2020	2021	2022	2023
1	Number of forensic examinations conducted, total:	130099	138042	124790	87603
	general fund	113202	114708	99083	70944
	special fund	16897	23334	25707	16659
2	Expenditures on forensic examinations, total:	11171.3	11992.2	11548.4	12614.3
	general fund	7939.0	8229.0	10170.4	8815.0
	special fund	3232.3	3763.3	1378.0	3799.3
3	Average cost of a forensic examination, total:	85.9	86.9	92.6	144.0
	general fund			102.7	124.3
	special fund			50.2	228.1
4	Number of scientific developments	129	118	128	110
	Number of completed developments	50	49	45	64
5	Expenditures on the development of forensic examination methods, total:	1398.5	1449.6	1753.3	1478.3
	general fund	1398.5	1449.6	1753.3	1478.3
6	Number of postgraduate and doctoral students	12	24	26	33
7	Training of highly qualified personnel, total:	5.8	13.6	11.6	21.7
	special fund	5.8	13.6	11.6	21.7
8	Number of purchased units of forensic equipment, total:		159	13	96
	general fund		81	12	2
	special fund		78	1	94
9	Purchase of forensic equipment, total:		3774.3	1280.6	1125.7
	general fund		1820.4	1258.1	199.7
	special fund		1953.9	22.5	925.9
10	Number of specialists taking advanced training, total:				1695
	special fund				1695
11	Average annual expenditures on advanced training of 1 specialist				0.04
12	Expenditures on professional development of specialists, total:				76.6
	special fund				76.6
13	Number of staff units, total:	1085	1084	1133	1163
	specialists (researchers, forensic experts)	941	943	982	1016
	technicians, laboratory assistants	36	22	20	18
	employees	102	111	121	119
	workers	6	8	10	10

In columns 2, 3, 5, 7, 9, 11 and 12, the amounts are in thousands of USD.

Compiled by the author on the basis of [33,37,41,45,52,53].

Attention should be paid to the annual increase in the number of forensic examinations in peacetime and a significant decrease after the full-scale invasion of Ukraine by the Russian Federation began.

The forensic research institutions of the Ministry of Justice of Ukraine conducted 397,937 forensic examinations (82.8 % of the total) at the request of law enforcement agencies, courts, etc. (general fund); and 82,597 examinations (17.2 % of the total) for citizens, lawyers, etc. (special fund), in particular, by year: in 2020, general fund – 87 %, special fund – 13 % of the total; in 2021, general fund – 83 %, special fund – 17 % of the total; in 2022, general fund – 79 %, special fund – 21 % of the total; in 2023, general fund – 81 %, special fund – 19 % of the total. There is an upward trend in the number of examinations carried out at the expense of individual requestors.

Since the military invasion of Ukraine, the average cost of one forensic examination has increased significantly: in 2021 compared to 2020 – by 1.01 times; in 2022 compared to 2021 – by 1.07 times; in 2023 compared to 2022 – by 1.56 times.

The Ministry of Justice of Ukraine justifies the increase in the cost of one forensic examination by the increase in the number of complex examinations due to the martial law and hostilities in the territory of Ukraine. The increase in the number of highly complex military, aviation, ballistic, and explosive examinations in criminal proceedings, which have gained wide publicity, has led to a significant increase in the workload of experts and an increased number of employees of forensic research institutions. 2023 saw an increase in the percentage of forensic examinations performed in accordance with international standards with the use of modern advances in science and technology and up-to-date forensic equipment.

The analysis of the information provided in the reports on the implementation of the budget programme passports has established a tendency of an increased receipt of funds for services provided by the research institutions of the Ministry of Justice of Ukraine. The cost of services is calculated by each forensic research institution of the Ministry of Justice of Ukraine separately [54], and the payment for provided services is transferred to the institution's account opened in the State Treasury of Ukraine.

In order to train highly qualified scientific and scientific-pedagogical personnel, the National Scientific Centre “Hon. Prof. M.S. Bokarius Forensic Science Institute” of the Ministry of Justice of Ukraine delivers postgraduate and doctoral courses in the speciality 081 Law with a forensic profile. Attention should be paid to the grown amount of in-service training of postgraduate and doctoral students, which is explained by the dissemination of information in society and the rising demand for this qualification.

The forensic research institutions of the Ministry of Justice of Ukraine ensure on a paid basis professional development for participants of short-term and long-term seminars: forensic experts, notaries, civil servants, insolvency receivers, realtors, and private enforcement officers [55].

The dynamics of the indicators - the number of scientific developments, the number of completed developments, and the costs of developing forensic examination methods – is due to the introduction of new types of examinations, which require the application of the available advanced research methods and creation of new ones.

The amount of expenditures for the purchase of forensic equipment at the expense of the special fund in 2023 increased due to an increase in the special fund's own revenues from rendering paid services by forensic research institutions, a subvention from the local budget, and forensic equipment donated by a charity organisation “European Charitable Traditions” and the Centre for Crisis Assistance and Support of the Ministry of Foreign and European Affairs of France.

It should be noted that when the State Budget of Ukraine is formed, the programme CPCEL 3601070 “Conducting forensic examinations and developing forensic examination methods” establishes the salary of forensic experts of forensic research institutions; however none of the reporting forms available on the website of the Ministry of Justice of Ukraine in the section ‘Financial Resources’ contains information on labour costs and social contributions of the state specialised institutions engaged in forensic activities.

The State Statistics Service of Ukraine publishes quarterly average monthly wages by type of economic activity: information and telecommunications, financial and insurance activities, education, healthcare and social assistance, etc [56]. Yet, there is no official statistical data on the average monthly salary in the field of forensic science, the organisational and managerial support of which is entrusted to six agencies – the Ministry of Justice of Ukraine, the Ministry of Health of Ukraine, the Ministry of Internal Affairs of Ukraine, the Ministry of Defence of Ukraine, the Security Service of Ukraine and the State Border Guard Service of Ukraine. This does not allow for an adequate analysis necessary to provide proposals for proper legislative regulation of remuneration of state forensic institutions’ employees. The absence of statistical data is evidence of the lack of research into this social problem [57].

For comparison, the U.S. Bureau of Labor Statistics website has a separate section dedicated to forensic experts, which contains information on professional employment and salaries, employment forecasts, education and work experience requirements, professional responsibilities, working conditions, etc. As an example, in May 2023, the average annual salary of forensic experts was USD 64,940, which was 1.4 times higher than the average monthly salary in the United States – USD 48,060 [58]. The following trend was observed: 10 % of forensic experts earned less than USD 41,410, while the remaining 10 % earned more than USD107,490 [59]. The employment rate in forensics in 2023 was 18,600 specialists. By 2033, it is projected to grow by 14 per cent to 20,200 specialists [60]. In the United States, state and local governments are expected to continue hiring forensic scientists to handle a large number of cases. Scientific and technological advances are predicted to increase the availability, reliability, and usefulness of objective forensic opinions used as evidence in courts, and therefore more forensic scientists will be needed to conduct research at the request of law enforcement agencies and courts.

The website of the Ministry of Justice of Ukraine does not contain information on financial activities by forensic research institutions. Out of the seven institutions of the Ministry of Justice of Ukraine, only Kyiv Scientific Research Institute of Forensic Expertise makes its financial statements publicly available by posting reports on financial results, cash flows, receipts and use of funds received as payment for services, etc. on its website in the section ‘Public Information’ (see Table 2).

**Table 2:** Financial results of Kyiv Scientific Research Institute of Forensic Expertise for the period 2020–2023.

№	Indicators	2020	2021	2022	2023
1	Budget allocations	3316298.0	3437433.6	4225682.7	3505607.6
2	Revenues from services (works) rendered	1103531.8	814474.5	380508.4	1134478.6
3	Income from sale of assets	55.2	158.4		
4	**Total revenues**	**4419880.0**	**4252063.2**	**4606191.1**	**4640086.2**
5	Labour costs and expenses	3344065.5	3758632.2	3612572.2	4143098.7
6	Contributions to social activities	710888.9	803419.6	789308.0	841790.1
7	Material costs	250826.4	254751.1	185579.2	288005.5
8	Depreciation and amortisation	486209.2	531010.3	453434.5	523900.1
9	Other expenses	15400.7	16680.6	1669.8	5327.5
10	Expenses on non-exchange transactions	657.7	2402.5	687.0	2222.7
11	**Total expenses**	**4808048.4**	**5366786.3**	**5043250.7**	**5804871.0**
12	Surplus/deficit for the reporting period	−388168.3	−1114723.1	−437059.7	−1164784.8

Amounts are stated in USD.

Compiled by the author on the basis of [33,37,41,45,[61], [62], [63], [64], [65], [66], [67], [68]].

Attention should be paid to a gradual increase in revenues for services provided by Kyiv Scientific Research Institute of Forensic Expertise despite the armed aggression of the Russian Federation against Ukraine: in 2020 – by 25 % of the total revenue; in 2021–19.2 %; in 2021–8.3 %; in 2022–24.5 %.

In 2021, the average number of employees of Kyiv Scientific Research Institute of Forensic Expertise was 384 [69], with an average monthly salary of USD 815.7, which was 1.7 times higher than the average monthly salary in Ukraine – USD 476.4 [37,70]; in 2022–381 persons [71] with the average monthly salary of USD 790.2, which exceeded the average monthly salary in Ukraine – USD 365.8 [41,72] by 2.2 times; in 2023–396 persons [73] with the average monthly salary of USD 871.9 exceeding the average monthly salary in Ukraine – USD 376.5 [45,74] by 2.3 times, respectively.

The salary of employees of state specialised institutions (non-military personnel and those who do not hold the rank of private or commander) who have the qualification of a forensic expert consists of a base salary, allowances, surcharges to it, bonuses and other payments, the amount and regulation of which are determined by the Cabinet of Ministers of Ukraine [1].

It should be noted that the Law of Ukraine “On Prevention of Corruption” equates experts for the purposes of this Law with persons authorised to perform the functions of the state or local self-government. However, experts are not listed as those obliged to submit a financial declaration of a person authorised to perform state or local government functions by April 1 yearly on the official website of the National Agency on Corruption Prevention [75]. The National Agency on Corruption Prevention controls, verifies, stores and discloses tax returns, and monitors the lifestyle of filers and their family members. At the same time, the National Agency on Corruption Prevention does not monitor the lifestyle of forensic experts who are not required to file an annual return.

In the context of the active implementation of numerous anti-corruption instruments and regulation of the legal basis for their application in Ukraine, the policy of the state of not including forensic experts in the list of persons obliged to file financial declarations is unclear.

## 4 Conclusions

An adequate level of financial support for forensic activities is the basis for fulfilling the tasks assigned to state expert institutions, observing the principles of legality, independence, objectivity and completeness of expertise, as well as a guarantee of the development of state expert institutions’ capacity, since this requires significant funds. A proper building of the financial support system for state forensic institutions and an optimal use of all financial sources are important factors of effective performance by the state of its main functions and tasks. An urgent objective is to search for reasonable opportunities to expand the financial autonomy of state specialised institutions and find ways to increase the efficiency of utilizing all available sources of their financial support.

Separate budget programmes of organization of activities and financing state specialised forensic institutions should be introduced for each ministry and other state bodies that are responsible for managing these institutions. This will resolve the issue of adequate funding, justification of expenditures, and will become an anti-corruption factor, for an open official declaration of incomes will prevent potential pressure as to distribution of funds from the political leadership on the heads of state specialised institutions and, accordingly, forensic experts.

Based on the results of the study, and taking into account the foreign experience, the author proposes a mixed system for determining the cost of forensic examination whereby: the cost of an expert hour is set by types and subtypes of forensic examinations; the number of sheets of a court case and additional documents to be examined is taken into account; three levels of complexity of an expert opinion are determined; and the list of actions for which an expert is entitled to a fixed remuneration, with its amount is fixed. In this case, the court should specify the type and subtype of forensic examination to be conducted, as well as determine the complexity of the forensic examination and the written opinion.

It is necessary to develop a unified methodological approach to calculating the number of examinations, focusing on ensuring the preparation of high-quality, complete, well-founded conclusions, rather than artificially increasing their number.

There is a need to summarise statistics on the financial support of forensic science in Ukraine, as this will facilitate effective administrative decisions. It is necessary to introduce the disclosure of financial statements to the public on the websites of the state specialised institutions that carry out forensic activities and on the websites of the relevant state agencies. When conducting the state statistical observation “Survey of Enterprises on Labour Statistics”, the average salary of the state specialised institutions engaged in forensic activities should be made public.

Given the specifics of their professional activities and the importance of their results for the administration of justice in Ukraine, forensic experts should be included in the list of persons obliged to submit an annual financial declaration on the official website of the National Agency on Corruption Prevention. However, control and verification of forensic experts' declarations, their storage and publication, and monitoring of the lifestyle of the declarants and their family members by the National Agency on Corruption Prevention do not imply total interference with either forensic experts’ private life or their professional activities.

The introduction of financial monitoring of forensic experts' lifestyles as an anti-corruption tool will improve the level of the domestic regulation of forensic activity, in particular with a focus on prevention of potential illegal actions, and will introduce effective mechanisms eliminating “artificial external” influence on the forensic expert's professional activities.

## Ethics approval and consent to participate

The given manuscript is a review article. Therefore, there is no requirement for ethical approval or participation consent. This article does not contain any studies with human participants or animals performed by the authors.

## Consent for publication

The author gives full consent for the publication of the article on approval.

## Availability of data and material

All required data pertaining to the manuscript has been already provided. There is no supplementary data.

## Funding

This research received no specific grant from any funding agency in the public, commercial, or not-for-profit sectors. This research received no external funding. This study was not funded.

## Declaration of competing interest

The authors declare that they have no known competing financial interests or personal relationships that could have appeared to influence the work reported in this paper.

## References

[1] Verkhovna Rada of Ukraine (1994). On forensic expertise: law of Ukraine on February 25, 1994 № 4038-XII. https://zakon.rada.gov.ua/laws/show/4038-12?lang=en#Text.

[2] Martynenko N. (2023). The system of public administration entities in the field of forensic expert activity: concept, status, powers, subordination and coordination. Int. J. Innov. Technol. Econo..

[3] Martynenko N. (2023). The state of research into the problems of public administration in the field of forensic expert activity. Public Admin. Region. Develop..

[4] Cabinet of Ministers of Ukraine (1996). On the approval of the Instructions on the procedure and amounts of compensation (reimbursement) of expenses and payment of remuneration to persons summoned to pre-trial investigation bodies, the prosecutor's office, the court or to the bodies in which cases of administrative offenses are pending, and payments to state specialized institutions of forensic examination for their execution by employees of the functions of experts and specialists: Decree of July 1, 1996 № 710. https://zakon.rada.gov.ua/laws/show/710-96-%D0%BF#Text.

[5] Lebedenko A. Yu (2023). Budgetary financing as the basis of financial support of forensic expert activity. Galician Econo. Bull..

[6] Khomutenko V.P., Khomutenko A.V. (2022). Conceptual principles of financial support of forensic expert activity in Ukraine. Scientific Notes of the Lviv University of Business and Law. Economic series. Legal series.

[7] Shunevich K.A. (2023). PHD Field of Knowledge 08 "Law": 12.00.08.

[8] Zhuravlyova M., Boimistruk A. (2023). Financial and material support of forensic expert activity: problematic and practical aspect. Word of the National School of Judges of Ukraine.

[9] Stashevska I.V., Martynova L.V. (2022). Implementation of the guarantee of the independence of the judicial expert in the context of his material and social security: the current state. Law and society. Criminal. Proced. Law Criminol..

[10] Shevchuk V.M. (2020). Problems of Reforming the Basic Legislation of Ukraine on Expert Provision of Justice: Materials of the Round Table.

[11] McAndrew W.P., Speaker P.J., Houck M.M. (2023). Interpol review of forensic management, 2019-2022. Forensic Sci. Int..

[12] Speaker P.J. (2024). Intelligence and the value of forensic science. Forensic Sci..

[13] Kaluzhna О. (2018). Politics or law: what is more in the approaches of public expert monopoly?. Problems of Legality.

[14] Gamette M. (2020). Improving forensic science integration: a Director's perspective. Forensic Sci. Int..

[15] McAndrew W.P., Houck M.M. (2020). Interpol review of forensic science management literature 2016-2019. Forensic Sci. Int..

[16] Bouzin J.T., Sauzier G., Lewis S.W. (2021). Forensic science in Seychelles: an example of a micro-jurisdiction forensic delivery system. Forensic Sci. Int..

[17] Howes L.M., Julian R., Wilson R., Dos Santos M.C. (2024). Forensic science capacity development: a case study of Timor-Leste. Forensic Sci. Int..

[18] Smith J.H., Horne J.S. (2024). Quality management system in forensic science: an African perspective. Forensic Sci. Int..

[19] Martynenko N., Domin D. (2023). Expert Assessments in Decision Making: Risks and Safety.

[20] Heavey A., Houck M.M., Turbett G.R., Lewis S.W. (2024). Quality issues in forensic science: a new perspective to support justice and innovation. Forensic Sci. Int.: Synergy.

[21] Castro A.E., De Ungria M.C.A. (2022). Methods used in microbial forensics and epidemiological investigations for stronger health systems. Forensic sciences research.

[22] Shonsey E. (2024). The benefits of a research and development sections in a state laboratory. Forensic Sci. Int.: Synergy.

[23] Cabinet of Ministers of Ukraine (2014). On the approval of the regulation on the ministry of justice of Ukraine: resolution № 228 dated july 2, 2014. https://zakon.rada.gov.ua/laws/show/228-2014-%D0%BF#Text.

[24] Verkhovna Rada of Ukraine (2010). Budget Code of Ukraine: Code of Ukraine dated july 8, 2010 № 2456-VI. https://zakon.rada.gov.ua/laws/show/2456-17#Text.

[25] Ministry of Justice of Ukraine (2024). Passports of budget programs. Financial resources. Areas of activity.

[26] Ministry of Justice of Ukraine (2020). About setting the cost of one expert hour in 2020. Nakaz dated January 30, 2020 № 328/5. https://zakon.rada.gov.ua/laws/show/z0109-20#Text.

[27] Ministry of Internal Affairs of Ukraine (2020). On establishing the normative cost of one expert hour at the main expert forensic center of the state border service of Ukraine in 2020: Nakaz № 302 of March 26, 2020. https://zakon.rada.gov.ua/laws/show/z0336-20#Text.

[28] Ministry of Internal Affairs of Ukraine (2020). On setting the normative cost of one expert hour in 2020: Nakaz № 255 of March 11, 2020. https://zakon.rada.gov.ua/laws/show/z0300-20#Text.

[29] National Bank of Ukraine (2020). Official exchange rate of the hryvnia against foreign currencies as of January 1, 2020. https://bank.gov.ua/ua/markets/exchangerates?date=01.01.2020&period=daily.

[30] Ministry of Justice of Ukraine (2021). On setting the cost of one expert hour in 2021: Nakaz dated January 29, 2021 № 343/5. https://zakon.rada.gov.ua/laws/show/z0144-21#Text.

[31] Ministry of Internal Affairs of Ukraine (2021). On establishing the normative cost of one expert hour at the main expert forensic center of the state Border service of Ukraine in 2021: Nakaz dated April 12, 2021 № 257. https://zakon.rada.gov.ua/laws/show/z0569-21#Text.

[32] Ministry of Internal Affairs of Ukraine (2021). On establishing the normative cost of one expert hour in the Expert Service of the Ministry of Internal Affairs of Ukraine in 2021: Nakaz dated March 29, 2021 № 227. https://zakon.rada.gov.ua/laws/show/z0506-21#Text.

[33] National Bank of Ukraine (2021). Official exchange rate of the hryvnia against foreign currencies as of January 1, 2021. https://bank.gov.ua/ua/markets/exchangerates?date=01.01.2021&period=daily.

[34] Ministry of Justice of Ukraine (2022). On setting the cost of one expert hour in 2022: order dated January 24, 2022 № 199/5. https://zakon.rada.gov.ua/laws/show/z0088-22#Text.

[35] Ministry of Internal Affairs of Ukraine (2022). On establishing the normative value of one export hour in the expert service of the ministry of internal Affairs of Ukraine in 2022: Nakaz of may 26, 2022 № 316. https://zakon.rada.gov.ua/laws/show/z0580-22#Text.

[36] Ministry of Internal Affairs of Ukraine (2022). On establishing the normative cost of one expert hour at the main expert forensic center of the state Border service of Ukraine in 2022: Nakaz dated June 7, 2022 № 342. https://zakon.rada.gov.ua/laws/show/z0623-22#Text.

[37] National Bank of Ukraine (2022). Official exchange rate of the hryvnia against foreign currencies as of January 1, 2022. https://bank.gov.ua/ua/markets/exchangerates?date=01.01.2022&period=daily.

[38] Ministry of Justice of Ukraine (2023). On setting the cost of one expert hour in 2023: Nakaz dated January 16, 2023 № 230/5. https://zakon.rada.gov.ua/laws/show/z0084-23#Text.

[39] Ministry of Internal Affairs of Ukraine (2023). On establishing the normative cost of one expert hour in the Expert Service of the Ministry of Internal Affairs of Ukraine in 2023: Nakaz dated March 15, 2023. https://zakon.rada.gov.ua/laws/show/z0546-23#Text.

[40] Ministry of Internal Affairs of Ukraine (2023). On establishing the normative cost of one expert hour at the main expert forensic center of the state Border service of Ukraine in 2023: Nakaz dated March 8, 2023 № 167. https://zakon.rada.gov.ua/laws/show/z0488-23#Text.

[41] National Bank of Ukraine (2023). Official exchange rate of the hryvnia against foreign currencies as of January 1, 2023. https://bank.gov.ua/ua/markets/exchangerates?date=01.01.2023&period=daily.

[42] Cabinet of Ministers of Ukraine (2023). On the introduction of amendments to paragraph 12 of the Instructions on the procedure and amounts of compensation (reimbursement) of expenses and payment of remuneration to persons summoned to pre-trial investigation bodies, the prosecutor's office, the court or to bodies in which cases of administrative offenses are pending, and payments to state specialized judicial institutions examinations for the performance by their employees of the functions of experts and specialists: Decree of December 1, 2023 № 1259. https://zakon.rada.gov.ua/laws/show/1259-2023-%D0%BF#Text.

[43] Ministry of Internal Affairs of Ukraine (2024). On establishing the normative cost of one expert hour in the Expert Service of the Ministry of Internal Affairs of Ukraine in 2024: Nakaz dated August 22, 2024 № 581. https://zakon.rada.gov.ua/laws/show/z1334-24#Text.

[44] Ministry of Internal Affairs of Ukraine (2024). On establishing the normative cost of one expert hour at the main expert forensic center of the state Border service of Ukraine in 2024: Nakaz of March 14, 2024 № 159. https://zakon.rada.gov.ua/laws/show/z0457-24#Text.

[45] National Bank of Ukraine (2024). Official exchange rate of the hryvnia against foreign currencies as of January 1, 2024. https://bank.gov.ua/ua/markets/exchangerates?date=01.01.2024&period=daily.

[46] Bundestag (2004). Gesetz über die Vergütung von Sachverständigen, Dolmetscherinnen, Dolmetschern, Übersetzerinnen und Übersetzern sowie die Entschädigung von ehrenamtlichen Richterinnen, ehrenamtlichen Richtern. Zeuginnen, Zeugen und Dritten (Justizvergütungs- und -entschädigungsgesetz - JVEG).

[47] European Commission for the Efficiency of Justice (2014). https://rm.coe.int/1680b044d2#P456_40972.

[48] Ministrstvo za pravosodje (2018). Pravilnik o sodnih izvedencih, sodnih cenilcih in sodnih tolmačih: PravilnikID: sprejeto: 20. 12. 2018 PRAV13490 EVA: 2018-2030-0025 SOP: 2018-01-4126. https://pisrs.si/pregledPredpisa?id=PRAV13490.

[49] Parlament České republiky (2019). Zákon o znalcích, znaleckých kancelářích a znaleckých ústavech: Zákon č. 254/2019 Sb. 10.09.2019. https://www.zakonyprolidi.cz/cs/2019-254.

[50] Ministerstvo spravedlnosti (2019). Vyhláška o znalečném: Vyhláška č. 504/2020 Sb. 07.12.2020. https://www.zakonyprolidi.cz/cs/2020-504.

[51] Cabinet of Ministers of Ukraine (2011). Some issues of the provision of paid services by scientific and research institutions of forensic examinations of the Ministry of Justice: resolution of July 27, 2011 № 804. https://zakon.rada.gov.ua/laws/show/804-2011-%D0%BF#Text.

[52] Ministry of Justice of Ukraine (2024). Results of the assessment of the effectiveness of budget programs for the reporting budget period. Areas of activity. Financial resources.

[53] Ministry of Justice of Ukraine (2024). Report on the execution of passports. Areas of activity. Financial resources. https://minjust.gov.ua/zvit_pro_vuk_passpots.

[54] Martynenko N. (2024). Areas of modernization of the system of training and certification of forensic experts in Ukraine based on the adaptation of foreign experience. Forensic Sci. Res..

[55] Kyiv branch of the National Research Center (2024). https://kyiv.nncise.org.ua/navchannya.

[56] State Statistics Service of Ukraine (2024). Average monthly salary by type of economic activity for the quarter. https://www.ukrstat.gov.ua/operativ/operativ2017/gdn/snzp/snzp_ek/smzp_ek_u.htm.

[57] Martynenko N. (2024). The mechanism for ensuring gender equality in the field of forensic expertise: a balanced gender policy. Balkan Soc. Sci. Rev..

[58] U. S. Bureau of Labor Statistics (2024). Occupational Outlook Handbook.

[59] U. S. Bureau of Labor Statistics (2024). Occupational Outlook Handbook.

[60] U. S. Bureau of Labor Statistics (2024). Occupational Outlook Handbook.

[61] (2020). Report on Financial Results for 2020.

[62] (2021). Report on Financial Results for 2021.

[63] (2022). Report on Financial Results for 2022.

[64] (2023). Kyiv research institute of forensic expertise of the ministry of justice of Ukraine. Report on financial results for 2023.

[65] (2020). Public Information. Report on the Use of Budget Funds. Report for 2020.

[66] (2021). Public Information. Report on the Use of Budget Funds. Report for 2021.

[67] (2022). Public Information. Report on the Use of Budget Funds. Report for 2022.

[68] (2023). Public Information. Report on the Use of Budget Funds. Report for 2023.

[69] (2021). Kyiv research institute of forensic expertise of the ministry of justice of Ukraine. https://m3rkxutm8pk3eqapl5ynjvb3gjps8uao.cdn-freehost.com.ua/wp-content/uploads/2023/01/form_f_d10.pdf.

[70] Pension Fund of Ukraine (2021). Average salary indicator for 2021. https://www.pfu.gov.ua/2130793-pokaznyk-serednoyi-zarobitnoyi-platy-za-2021-rik/.

[71] (2022). Kyiv research institute of forensic expertise of the ministry of justice of Ukraine. https://m3rkxutm8pk3eqapl5ynjvb3gjps8uao.cdn-freehost.com.ua/wp-content/uploads/2023/06/form_f_d10-1.pdf.

[72] Pension Fund of Ukraine (2022). Average salary indicator for 2022. https://www.pfu.gov.ua/2152284-pokaznyk-serednoyi-zarobitnoyi-platy-za-2022-rik/.

[73] (2023). Kyiv research institute of forensic expertise of the ministry of justice of Ukraine. https://m3rkxutm8pk3eqapl5ynjvb3gjps8uao.cdn-freehost.com.ua/wp-content/uploads/2024/02/form_f_d10.pdf.

[74] Pension Fund of Ukraine (2023). Average salary indicator for 2023. https://www.pfu.gov.ua/2158510-pokaznyk-serednoyi-zarobitnoyi-platy-za-2023-rik/.

[75] Verkhovna Rada of Ukraine (2014). https://zakon.rada.gov.ua/laws/show/1700-18#Text.

